# Revision of “*Phyllobrotica*” from Taiwan with description of *Jolibrotica* gen. n. (Coleoptera, Chrysomelidae, Galerucinae)

**DOI:** 10.3897/zookeys.547.9381

**Published:** 2015-12-17

**Authors:** Chi-Feng Lee, Jan Bezděk

**Affiliations:** 1 Applied Zoology Division, Taiwan Agricultural Research Institute, 189 Chung-Cheng Road, Wufeng, Taichung 413, Taiwan; 2 Department of Zoology, Mendel University, Zemědělská 1, 613 00 Brno, Czech Republic

**Keywords:** *Jolibrotica* gen. n., *Haplosomoides*, Verbenaceae, taxonomic revision, Palaearctic Region, new genus

## Abstract

All Taiwanese species formerly classified the genus *Phyllobrotica* Chevrolat, 1836 are revised. *Jolibrotica* Lee & Bezděk, **gen. n.**, is described for *Phyllobrotica sauteri* (Chûjô, 1935) (Taiwan, China: Guangxi) and *Phyllobrotica chujoi* Kimoto, 1969 (Taiwan). *Phyllobrotica shirozui* Kimoto, 1969 is transferred to the genus *Haplosomoides*. All species are redescribed and their diagnostic characters illustrated.

## Introduction

*Luperus sauteri* Chûjô was described in 1935 and named in honor of Hans Sauter. Later Chûjô (1963) described another species in honor of this German entomologist, *Phyllobrotica sauteri* Chûjô. Kimoto (1969) transferred the older species to *Phyllobrotica* and proposed a new replacement name, *Phyllobrotica chujoi* Kimoto, 1969 for more recent species due to homonymy. A third *Taiwanese Phyllobrotica* species, *Phyllobrotica shirozui* Kimoto, 1969 was described by himin the same paper.

Currently, the genus *Phyllobrotica* Chevrolat, 1836 is composed of 12 species from the Palaearctic, 2 from the Oriental, and 17 from the Nearctic Region (Bezděk 2010). However, the three Taiwanese species differ from *Phyllobrotica* in important characteristics. One of these, *Phyllobrotica shirozui* Kimoto, 1969, actually belongs to *Haplosomoides* Duvivier, 1890. The two remaining species, *Phyllobrotica sauteri* (Chûjô, 1935) and *Phyllobrotica chujoi* Kimoto, 1969, need to be classified in a new genus, *Jolibrotica* gen. n., described here in.

Based on reduced elytral epipleurae the genus *Jolibrotica* gen. n. should be classified in the section Phyllobrotices of Luperina (Luperini). This section was proposed by Chapuis (1875) exclusively for genera with reduced epipleurae. Wilcox (1965) stated that the section Phyllobrotices was poorly and tentatively defined. Surprisingly, Wilcox (1973) combined the sections Phyllobrotices and Mimastrites, the latter containing genera with well developed epipleurea, which made the definition of the section Phyllobrotices even more ambivalent. The same arrangement was used in the generic list of Seeno and Wilcox (1982). It is also necessary to note that there is a lack of modern phylogenetic studies on Luperini system. The arrangement of various sections within Luperini should be revised in the future.

Both species of *Jolibrotica* gen. n. were previously placed in *Phyllobrotica* based on the reduced epipleurae. However, the genus *Phyllobrotica* is completely different from any species from Taiwan (see Diagnosis below). *Jolibrotica* gen. n. is known from Taiwan and several females tentatively assigned to *Jolibrotica sauteri* were collected also in continental China (Guangxi).

The Taiwan Chrysomelid Research Team (TCRT) was founded in 2005 and is composed of 10 members. Most of them amateurs interested in making an inventory of all species of Chrysomelidae in Taiwan. Specimens of the new genus have been extensively surveyed and studied, and host plants recorded. Diagnostic characters were assessed and the status of all species was evaluated based on a series of more than 400 specimens. Most of them were collected by the TCRT and others belonged to the historic collection of TARI.

## Materials and methods

To prepare drawings of the adult reproductive systems, the abdomens of adults were separated and boiled in a 10% KOH solution, cleared in distilled water, and then mounted on microscope slides in glycerin for observation. Specimens were examined and drawings were made using a Leica M165 stereomicroscope. Microscope slides were examined and illustrated using a Nikon ECLIPSE 50i microscope. Body parts were then stored in glycerin tubes with the dry mounted specimens.

Host plants are recorded by observing adult feeding behavior in the field.

Specimens examined are deposited at the following institutes and museums.

BMNH The Natural History Museum, London, UK [Michael Geiser];

NHM Hungarian Natural History Museum, Budapest, Hungary [Ottó Merkl];

JBCB Jan Bezděk collection, Brno, Czech Republic;

KUEC Faculty of Agriculture, Kyushu University, Fukuoka, Japan [Osamu Tadauchi];

KMNH Kitakyushu Museum of Natural History, Kitakyushu, Japan [Yûsuke Minoshima];

NMPC National Museum, Prague, Czech Republic [Jiří Hájek];

SDEI Senckenberg Deutsches Enomologisches Institut, Müncheberg, Germany [Stephan Blank];

TARI Taiwan Agricultural Research Institute, Taichung, Taiwan

Exact label data are cited for all type specimens of the described species; a double slash (//) divides the data on different labels and a single slash (/) divides the data in different rows. Other comments and remarks are in square brackets: [p] – preceding data are printed, [h] – preceding data are handwritten, [w] – white label, [y] – yellow label, [b] – blue label, and [r] – red label.

## Taxonomy

### Jolibrotica gen. n.

Taxon classificationAnimaliaColeopteraChrysomelidae

http://zoobank.org/A2B1F043-F232-49A4-A565-9556016BA172

#### Type species.

Luperus (Luperus) sauteri Chûjô, 1935

#### Description.

Coloration: dorsum lustrous, black or metallic blue-green. Antennae black. Legs metallic,black, or brown. Ventral side metallic or black. Body length 3.2–4.3 mm.

Head. Labrum trapezoidal, transverse, with four pores in transverse row bearing pale seta, anterior margin straight. Anterior part of head very short, almost impunctate and glabrous, several setae on anterior margin of clypeus and along lateral margins of nasal keel. Nasal keel narrow, sharp. Interantennal space very narrow, cca 0.5 as wide as transverse diameter of antennal insertion. Frontal tubercles transverse, subtriangular, slightly elevated, lustrous, glabrous, impunctate, anterior tips not separated by nasal keel. Vertex with distinct shallow impression in middle just behind frontal tubercles, with several larger punctures at each side just behind frontal tubercles bearing very long pale setae, rest of vertex impunctate or with indistinct fine punctuation and glabrous. Antennae slender, 0.80–1.00 as long as body, all antennomeres dull, covered with dense setae, antennomere II as long as wide, antennomere III three times as long as antennomere II, antennomeres III-VII ca 2.8–3.0 as long as wide.

Pronotum 1.30–1.60 times as broad as long, widest in anterior quarter, parallel anteriorly, convergent posteriorly, anterior margin straight, posterior margin rounded. Disc covered with fine punctures. Posterior half of disc with wide shallow transvese impression. Anterior margin unbordered in middle, laterally with indistinct fine border, lateral and posterior margins bordered. Anterior and posterior margins with dense short setae, lateral margins with sparse setae. Anterior angles moderately swollen, recangular, posterior angles obtuseangulate, all angles with setigerous pore bearing long pale seta.

Scutellum subtriangular, impunctate, glabrous, with rounded apex.

Elytra ca 1.90–2.10 times as long as wide, almost glabrous (with almost indistinct very scarse short pale setae on humeri, lateral margins and apical slopes), widest at apical quarter, densely covered with fine small confused punctures. Humeral calli well developed. Epipleura extremely narrow, visible only in anterior third of elytra, towards apex more or less only indicated. Macropterous.

Ventral surface lustrous, sparsely covered with fine punctures and pale setae. Anterior coxal cavities opened posteriorly. Prosternal process not visible between procoxae. Abdomen simple, posterior margin of last ventrite with two short incisions, surface behind posterior margin subtriangularly impressed.

Legs slender. All tibiae with fine apical spine in both sexes. Protarsomeres I slender, ca 0.75 times as long as II and III combined. Metatarsomeres I slender, ca as long as II and III combined. Claws appendiculate.

Penis (Figs 9, 10, 25, 26) extremely elongate, without lateral processes, weakly curved at lateral view; internal sac with at least one elongate sclerite.

Females. Antennae distinctly more slender than in males. Protarsomeres I same as in males. Posterior margin of last ventrite entire. Gonocoxae (Figs 11, 27) slender, well separated from each other, narrowly connected at middle; each gonocoxa with seven setae from apical 1/6 to apex. Ventrite VIII (Figs 12, 26) well sclerotized; apical margin widely rounded, with dense setae along outer margin. Spermatheca very characteristic, spermathecal receptaculum (Fig, 13, 29) extremely swollen; pump extremely slender and curved; sclerotized spermathecal duct short and wide, hardly separated from receptaculum.

#### Diagnosis.

*Jolibrotica* gen. n. can be differentiated from *Phyllobrotica* as follows: body black or metallic; interantennal space very narrow, cca 0.5 as wide as transverse diameter of antennal insertion; vertex with several larger punctures at each side just behind frontal tubercles bearing very long pale setae; antennae 0.80–1.00 as long as body; antennomere II as long as wide, antennomere III three times as long as II; male abdominal ventrites not modified; all tibiae with fine apical spine in both sexes; body length 3.2–4.3 mm. Same characters in *Phyllobrotica*: body coloration always partly orange; interantennal space wider, ca as wide as transverse diameter of antennal insertion; vertex completely glabrous except one setigerous pore behind each eye; antennae distinctly shorter than body; antennomere II ca twice as long as wide, antennomere III 1.5 times as long as II; male abdominal ventrites strongly modified; all tibiae in both sexes without apical spines; body length more than 5.0 mm.

Based on Wilcox (1973) and Seeno and Wilcox (1982), the section Phyllobroticites includes the following Asiatic genera: *Euliroetis* Ogloblin, 1936; *Japonitata* Strand, 1935; *Hoplasoma* Jacoby, 1884 (= *Haplomela* Chen, 1942); *Hemygascelis* Jacoby, 1896; *Konbirella* Duvivier, 1892; *Mimastra* Baly, 1865 (? = *Neoatysa* Abdullah & Qureshi, 1968); *Trichomimastra* Weise, 1922; *Haplosomoides* Duvivier, 1890; *Sosibiella* Jacoby, 1896. All these genera (except *Konbirella*) can be easily distinguished from *Jolibrotica* gen. n. by tibiae lacking apical spines. Additional distinguishing characters can be described as follows: *Euliroetis* has the male abdomen strongly modified and penis bifurcate (abdomen not modified in male and penis extremely elongate, not bifurcate in *Jolibrotica* gen. n.), *Japonitata* has elytra bearing distinct carinae and impressions (elytra even in *Jolibrotica* gen. n.), *Hoplasoma* has bifurcate claws (claws appendiculate in *Jolibrotica* gen. n.), *Hemygascelis* has the male abdomen strongly modified and pronotum much longer than wide (abdomen simple and pronotum transverse in *Jolibrotica* gen. n.), *Mimastra* has epipleurae that are wide in the basal quarter, than suddenly narrowed and visible towards apex (epipleurae extremely narrow, visible only in anterior third of elytra in *Jolibrotica* gen. n.), *Trichomimastra* has the elytra densely pubescent (almost glabrous in *Jolibrotica* gen. n.), *Haplosomoides* is larger and the body is completely or predominantly yellow (body smaller and completely black or metallic in *Jolibrotica* gen. n.), and, finally, *Sosibiella* has wide epipleurae. *Konbirella* and *Jolibrotica* gen. n. apparently are the only genera in Phyllobroticites with apical spurs on all tibiae. *Konbirella* differs by possessing antennae that are 1.3 times longer than the body and the pronotum is longer than wide (antennae shorter, 0.80–1.00 as long as body and pronotum transverse in *Jolibrotica* gen. n.).

Recently, three additional genera were described and probably belong to Phyllobroticites although it is not specified in the description. From *Jolibrotica* gen. n. they can be separated as follows: *Pubibrotica* Medvedev, 2002 has tibiae lacking apical spines and elytra densely pubescent; *Mimastrosoma* Medvedev, 2004 is much larger, predominantly pale and the aedeagus is bifurcate; *Hirtomimastra* Medvedev, 2009 has elongate metatarsus I, body pale and elytra densely pubescent.

#### Distribution.

Taiwan, China: Guanxi.

#### Etymology.

Composed from Jolivet and *Phyllobrotica* to honor Pierre Jolivet, who promoted leaf beetle research more than any other person in recent history.

### Jolibrotica sauteri

Taxon classificationAnimaliaColeopteraChrysomelidae

(Chûjô, 1935) comb. n.

Luperus (Luperus) sauteri Chûjô, 1935: 162.
Luperus sauteri : Chûjô, 1962: 238.
Phyllobrotica sauteri : Kimoto, 1969: 38; Wilcox 1973: 474; Kimoto 1989: 252; Kimoto and Chu 1996: 72; Kimoto and Takizawa 1997: 303, 377; Beenen 2010: 487.

#### Type locality.

Taiwan: New Taipei City, Tinshungchi [頂雙溪] (= Chosokei), 25°01'27"N, 121°52'22"E, 50 m.

#### Type material examined.

Lectotype male (TARI), pinned, here designated to fix the concept of Luperus (Luperus) sauteri Chûjô and to ensure the universal and consistent interpretation of the same, labeled: “Chosokei [= Tinshungchi, New Taipei City] (Form) / H Sauter, 1914 [p, w] // COTYPE [p, circle label with yellow letters] // *Luperus* / *sauteri* / Chûjô [h] / DET. M. CHUJO [p, b] // No. 1356 [p, w] // **Lectotypus** / *Luperus sauteri* ♂ / Chûjô, 1935/ des. C.-F. Lee, 2015 [p, r]”. Paralectotypes: 1♀ (TARI): “Chosokei (Form) / H Sauter, 1914 [p, w] // COTYPE [p, circle label with yellow letters] // *Luperus* / *sauteri* / Chûjô [h] / DET. M. CHUJO [p, b] // No. 1355 [p, w]”; 1♂, 1♀ (SDEI): “Chosokei (Form) / H Sauter, 1914 [p, w] // Syntypus [p, r] // *Luperus* / *sauteri* / Chûjô [h] / DET. M. CHUJO [p, b] // DEI Müncheberg / Col – 05057 and 05058”; 1♂ (SDEI): “Taihoku-Dist. / Maruyma [= Yuanshan, Taipei City] XII.1912 [p, w] // Syntypus [p, r] // *Luperus* / *sauteri* / Chûjô [h] / DET. M. CHUJO [p, b] // DEI Müncheberg / Col – 05056”. Each paralectotype has a type label: “**Paralectotypus** [p] / *Luperus sauteri* ♂ [or ♀] [p] / Chûjô, 1935 [p] / des. C.-F. Lee, 2015 [p, pink label]”

#### Additional specimens examined

(n = 181). **TAIWAN. Chiayi**: 1♂, 1♀, Lachitashan, 19.III.2009, leg. H. Lee (TARI); 2♀♀, Tutzuhu trail, 1.VI.2014, leg. W.-C. Liao (TARI); **Hsinchu**: 1♂, 1♀, Litungshan, 26.II.2009, leg. S.-F. Yu (TARI); 4♂♂, 15.III.2009, leg. M.-H. Tsou (TARI); 2♂♂, 3♀♀, same locality, 13.III.2009, leg. M.-H. Tsou (TARI); 1♂, Lupi, 4.IV.2009, leg. M.-H. Tsou (TARI); 1♀, Talu logging trail, 24.VI.2009, leg. Y.-F. Hsu (TARI); **Hualien**: 14♂♂, 8♀, coastal range SE of Fuli, 12.-16.XI.2008, leg. L. Dembický (BMNH, 2♂♂, 1♀ JBCB);. **Ilan**: 1♀, Chiaosi, 7.XII.2008, leg. H.-J. Chen (TARI); 1♀, Fushan Botanical Park, 14.II.2009, leg. M.-H. Tsou (TARI); 3♂♂, 2♀♀, same locality, 20.III.2009, leg. C.-F. Lee (TARI); 1♂, Hsinliao, 19.I.2010, leg. S.-F. Yu (TARI); 1♀, Mingchi, 27.IV.2008, leg. M.-H. Tsou (TARI); 1♀, Fushan Chihwuyan, 20.III.2009, leg. C.-F. Lee (JBCB); **Kaoshiung**: 3♂♂, Chungchihkuan, 16–17.IV.2012, leg. L.-P. Hsu (TARI); 1♂, 2♀♀, Chuyunshan, 1.III.2009, leg. U. Ong (TARI, 1♀ JBCB); 3♂♂, 24.III.2009, leg. C.-F. Lee (TARI); 1♂, 5♀♀, Shihshan logging trail, 24.III.2009, M.-H. Tsou (TARI); 1♂, 8♀♀, Tengchih, 2–5.VI.2008, leg. C.-F. Lee (TARI, 1♂, 1♀ JBCB); 2♂♂, 4♀♀, same locality, 26.V.2009, leg. C.-F. Lee (TARI); 1♀, same locality, 4.VIII.2012, leg. J.-C. Chen (TARI); 1♂, Tona trail, 3.II.2013, leg. B.-X. Guo (TARI); 1♀, same locality, 3.II.2013, leg. W.-C. Liao (TARI); 1♂, 2♀♀, same locality, 9.XI.2013, leg. W.-C. Liao (TARI); **Keelung**: 1♀, Lungkang trail, 5.IV.2011, leg. H. Lee (TARI); 1♀, Tawulunshan, 21.III.2009, leg. H.-J. Chen (TARI); **Nantou**: 1♂, 2♀♀, Lushan, 7.III.2009, leg. U. Ong (TARI); 3♂♂, Meifeng, 19–21.IV.1983, leg. K. C. Chou & S. P. Huang (TARI); 1♂, Peitungyanshan, 14.V.2014, leg. C.-F. Lee (TARI); 4♂♂, 2♀♀, Tatachia, 9.VI.2009, leg. C.-F. Lee (TARI); 1♂, Tsuifeng, 23.V.1982, leg. L. Y. Chou (TARI); 1♂, Tungpu, 20–24.VI.1983, leg. K. C. Chou & C. Y. Wong (TARI); 2♀♀, same locality, 16–20.IV.1984, leg. K. C. Chou & C. H. Yung (TARI); 1♀, Tunyuan, 10.III.2010, leg. Y.-F. Hsu (TARI); 13♂♂, 12♀♀, Wushe, 19–22.IV.1983, leg. K. C. Chou & S. P. Huang (TARI); **Pingtung**: 2♀♀, Lilungshan, 23.XII.2009, leg. J.-C. Chen (TARI); 2♀♀, Peitawushan, 17.II.2010, leg. M.-H. Tsou (TARI); 1♂, same locality, 19.II.2014, leg. Y.-T. Chung (TARI); 1♀, Tahanshan, 6.II.2008, leg. S.-F. Yu (TARI); 1♂, 3.III.2008, leg. C.-F. Lee (TARI); 1♀, same locality, 25.V.2008, leg. C.-F. Lee (TARI); 1♂, same locality, 21.III.2009, leg. M.-H. Tsou (TARI); 1♀, same locality, 5.IV.2009, leg. C.-F. Lee (TARI); 1♀, 6.IV.2013, leg. W.-C. Liao (TARI); 1♂, same locality, 19.II.2014, leg. Y.-T. Chung (TARI); **Taichung**: 2♂♂, Kukuan, 19.III.2014, leg. C.-F. Lee (TARI); 1♀, Tahsuehshan, 24.IV.2012, leg. C.-F. Lee (TARI); 3♂♂, 5♀♀, Wushihkeng, 19.III.2008, leg. C.-F. lee (TARI, 1♂ JBCB); **Taipei**: 1♀, Fushan, 2.III.2012, leg. H.-J. Chen (TARI); 1♂, Hsiaoyukeng, 29.III.2008, leg. M.-H. Tsou (TARI); 1♂, 5♀♀, Sukanshui, 24.XII.2006, leg. S.-F. Yu (TARI); 1♀, Tanshui, 9.IV.2008, leg. W.-T. Liu (TARI); 3♀♀, same locality, 19.IV.2009, leg. H.-T. Cheng (TARI); 2♀♀, Wulai, 3.XII.2006, leg. M.-H. Tsou (TARI); 1♀, same locality, 22.XII.2006, leg. H.-J. Chen (TARI); 3♂♂, 3♀♀, same locality, 28.II.2007, leg. C.-F. Lee (TARI, 1♂ JBCB); 1♂, same locality, 22.II.2008, leg. H.-J. Chen (TARI); 3♂♂, same locality, 2.I.2010, leg. H. Lee (TARI); 1♂, same locality, 21.II.2010, leg. Y.-L. Lin (TARI); 1♀, same locality, 17.III.2010, leg. H.-J. Chen (TARI); 1♀, same locality, 17.III.2010, leg. C.-F. Lee (TARI); 1♂, 2♀♀, Yangmingshan, 15.III.1998, leg. C.-F. Lee (TARI); 1♀, same locality, 3.V.2009, leg. M.-H. Tsou (TARI); **Taitung**: 2♀♀, Lichia, 15–16.VII.2014, leg. Y.-T. Chung (TARI); 1♀, Liyuan, 19.IV.2014, leg. W.-C. Huang (TARI); **Taoyuan**: 1♀, Fuhsing, 6.V.1983, leg. K. C. Chou & C. C. Pan (TARI); 2♂♂, Hsuehwunao, 2–3.IV.2011, leg. M.-H. Tsou (TARI); 1♀, same locality, 10.IV.2011, leg. M.-H. Tsou (TARI); 1♂, Lalashan, 2.IV.2009, leg. C.-F. Lee (TARI); 3♀♀, Paling, 3–5.V.1983, leg. K. C. Chou & C. C. Pan (TARI). **CHINA. Guangxi**: 5♀♀, Longsheng Hot Spring, 25°53.6´N 110°12.4´E, 360 m, 11.-14.iv.2013, M. Fikáček, J. Hájek & J. Růžička leg. (NMPC).

#### Diagnosis.

*Jolibrotica sauteri* is characterized by its metallic blue or green color and extremely elongate penis.

#### Males.

**Figures 1–6.**
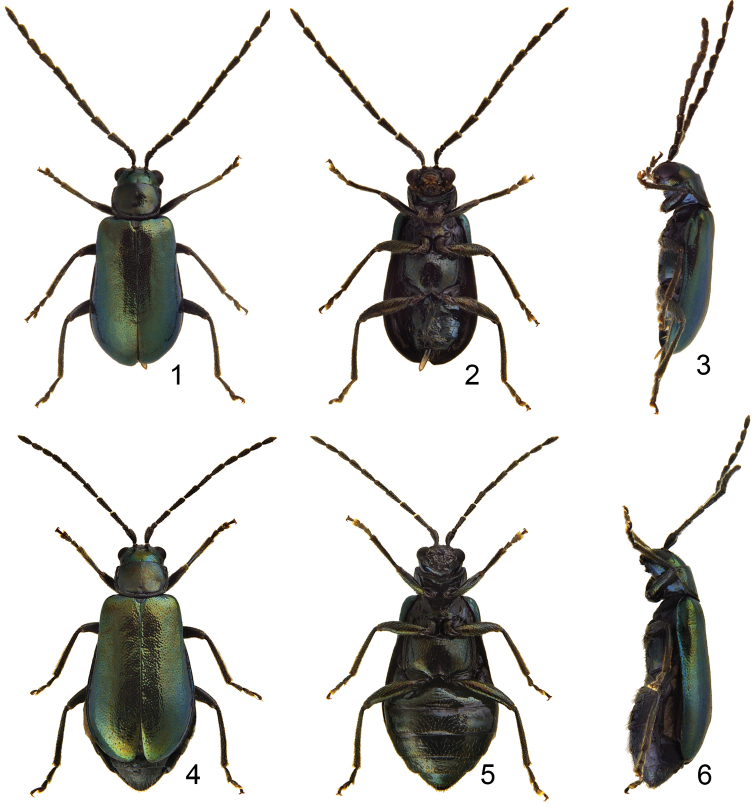
Habitus of *Jolibrotica sauteri*. **1** Male, dorsal view **2** ditto, ventral view **3** ditto, lateral view **4** Female, dorsal view **5** ditto, ventral view **6** ditto, lateral view.

**Figures 7–13.**
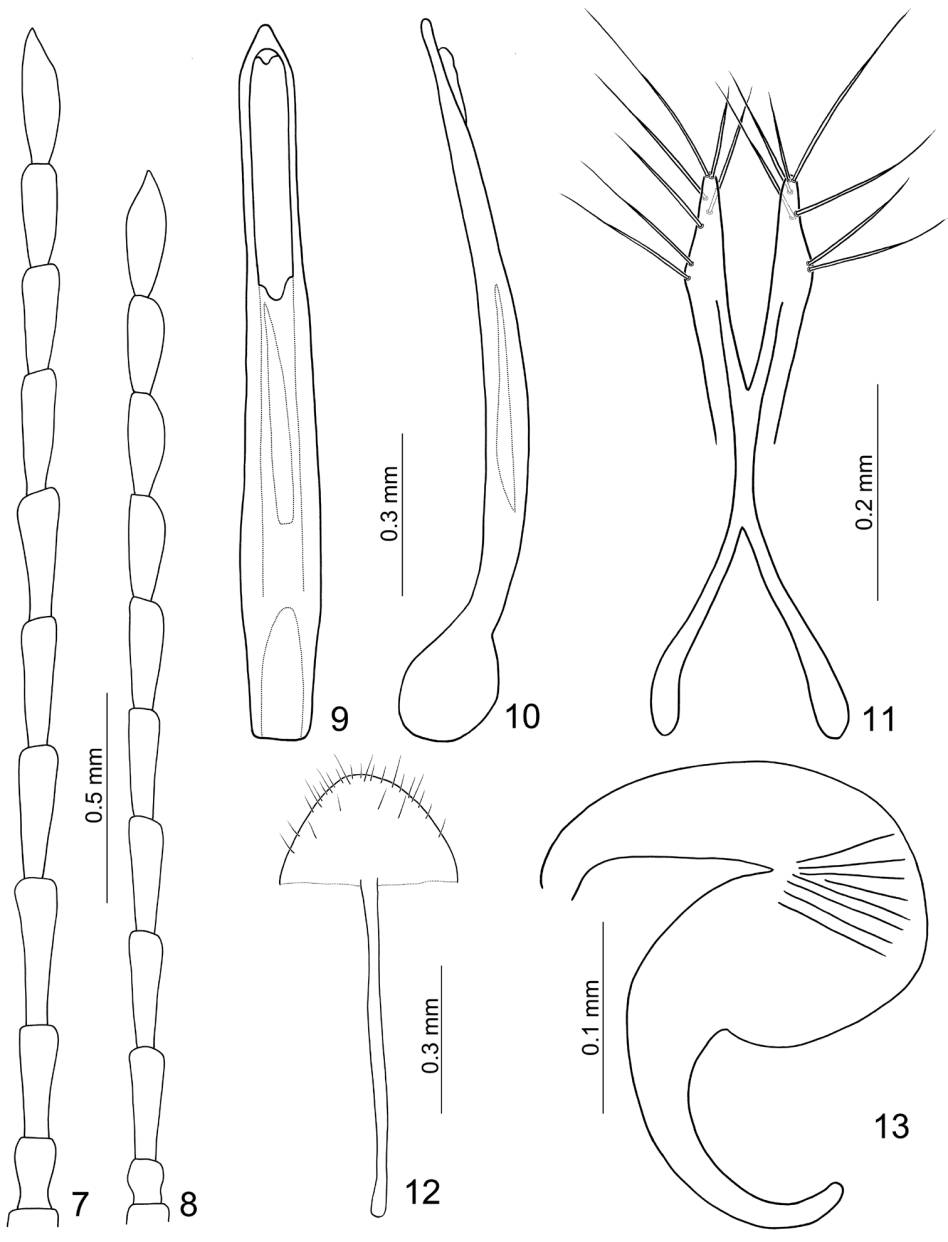
*Jolibrotica sauteri*. **7** Antenna male **8** Antenna, female **9** Penis, dorsal view **10** Penis, lateral view **11** Gonocoxae **12** Ventrite VIII **13** Spermatheca.

Length 3.3–3.8 mm, width 1.4–1.6 mm. Color metallic green or blue (Figs 1–3); antenna and legs black. Eyes small, distance between eyes 3.0 times wider than diameter of eye. Antenna (Fig. 7) filiform and long, as long as body, ratio of length of antennomeres III to XI about 1.0 : 1.0 : 1.0 : 1.0 : 1.0 : 1.0 : 1.0 : 1.0 : 1.3; ratio of length to width from antennomere III to XI about 2.6 : 2.7 : 2.6 : 2.6 : 2.6 : 2.6 : 2.0 : 2.0 : 4.0. Pronotum quadrilateral; 1.43–1.48 times wider than long; widened anteriorly; disc moderately depressed behind middle. Elytra elongate, 1.73-1.78 times longer than wide; widest at apical 1/3. First tarsomeres normal. Abdominal ventrites without modification, ventrite V with apical margin truncate. Penis (Figs 9, 10) extremely elongate, about 8.8 times longer than wide; parallel-sided, slightly wider in basal third; tectum membranous; ventral surface with longitudinal median area membranous; extremely slender and slightly curved in lateral view; internal sac with one longitudinal sclerite, apically pointed and with base truncate.

#### Females.

Length 3.6–3.9 mm, width 1.8–1.9 mm. Similar to male (Figs 4–6); antenna relatively shorter and slender than male (Fig. 8), about 0.8 times as long as body, ratio of length of antennomeres III to XI about 1.0 : 1.0 : 1.0 : 1.0 : 1.0 : 0.9 : 0.9 : 0.8 : 1.2; ratio of length to width from antennomere III to XI about 3.3 : 3.3 : 3.3 : 3.3 : 3.3 : 2.9 : 2.8 : 2.5 : 3.2. Apical margin of ventrite V truncate. Gonocoxae (Fig. 11) slender, well separated from each other, combined together from apical 2/5 to 3/5; each gonocoxa with seven setae from apical 1/6 to apex. Ventrite VIII (Fig. 12) well sclerotized; apical margin widely rounded, with several long setae near lateral margins, and several long and short setae mixed along apical margin. Spermathecal receptaculum (Fig. 13) extremely swollen; pump extremely slender and curved; sclerotized spermathecal duct short and wide.

#### Distribution.

**Figures 14–16.**
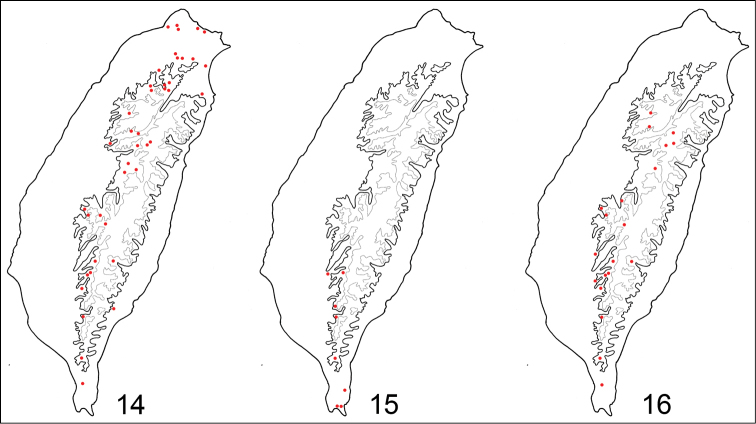
Distribution maps, solid line: 1000 m, broken line: 2000 m. **14** *Jolibrotica sauteri* **15** *Jolibrotica chujoi* **16** *Haplosomoides shirozui*.

Taiwan, China: Guangxi. *Jolibrotica sauteri* is more widespread (Fig. 14) than *Jolibrotica chujoi* (Fig. 15). In Taiwan, most adults appear below 1500 m elevation and are active during winter.

#### Host plant.

Callicarpa formosana Rolfe var. formosana Rolfe (Verbenaceae).

#### Comments.

Four females collected in Guangxi are tentatively assigned to *Jolibrotica sauteri*. No difference were observed between females from Guangxi and Taiwan. The shapes of spermatheca and ventrite VIII of females from Guangxi slightly differ from Taiwan specimens, but such slight differencies may be infraspecific. The gonocoxae from both populations are identical.

### Jolibrotica chujoi

Taxon classificationAnimaliaColeopteraChrysomelidae

(Kimoto, 1969) comb. n.

Phyllobrotica sauteri Chûjô, 1963: 395.
Phyllobrotica chujoi Kimoto, 1969: 38 (replacement name); Wilcox 1973: 471; Kimoto 1989: 252; Kimoto 1991: 12; Kimoto and Chu 1996: 72; Kimoto and Takizawa 1997: 303, 377; Beenen 2010: 487.

#### Type locality.

Taiwan: Kaoshiung city, Chiasien [甲仙] (= Kosempo), 23°06'52"N, 120°37'53"E, 500 m.

#### Type material examined.

Holotype ♂ (HNHM), labeled: “Kosempo [= Chiasien, in Kaoshiung] / 980. [p, w] // Formosa / Sauter [p, w] // Holotype [h, r] // Holotypus [p, red letters] / Phyllobrotica / sauteri / Chujo [h, w, with red border] // *Phyllobrotica* / *sauteri* Chûjô [h] / Det. M. CHUJO, 1961 [p, w]”.

#### Additional specimens examined

(n = 21). **TAIWAN. Kaoshiung**: 1♀, Tengchih, 4.VII.2011, leg. M.-H. Tsou (TARI); 1♀, same locality, 8.VI.2013, leg. W.-C. Liao (TARI); **Pingtung**: 1♂, Kenting, 22-26.III.1982, leg. T. Lin & S. C. Lin (TARI); 3♂♂, 2♀♀, Nanjenhu, 31.III.2011, leg. J.-C. Chen (TARI, 1♂, 1♀ JBCB); 1♂, Peitawushan, 8.V.2014, leg. J.-C. Chen (TARI); 1♀, same locality, 3.VI.2014, leg. Y.-T. Chung (TARI); 3♂♂, Sheding Park, 9.IV.2012, leg. Y.-C. Lan & W.-N. Lu (TARI, 1♂ JBCB); 1♀, Sheting, 5.V.2010, leg. J.-C. Chen (JBCB); 1♂, Wutai, 22.III.2010, leg. J.-C. Chen (TARI); 2♂♂, 3♀♀, same locality, 23.VI.2014, leg. J.-C. Chen (TARI); 1♂, 8-15 km NE of Hengchung, 15.-20.VI.2008, leg. F. & L. Kantner (JBCB).

#### Diagnosis.

*Jolibrotica chujoi* is similar to *Jolibrotica sauteri* but differs by its shiny black color and wider penis.

#### Males.

**Figures 17–22.**
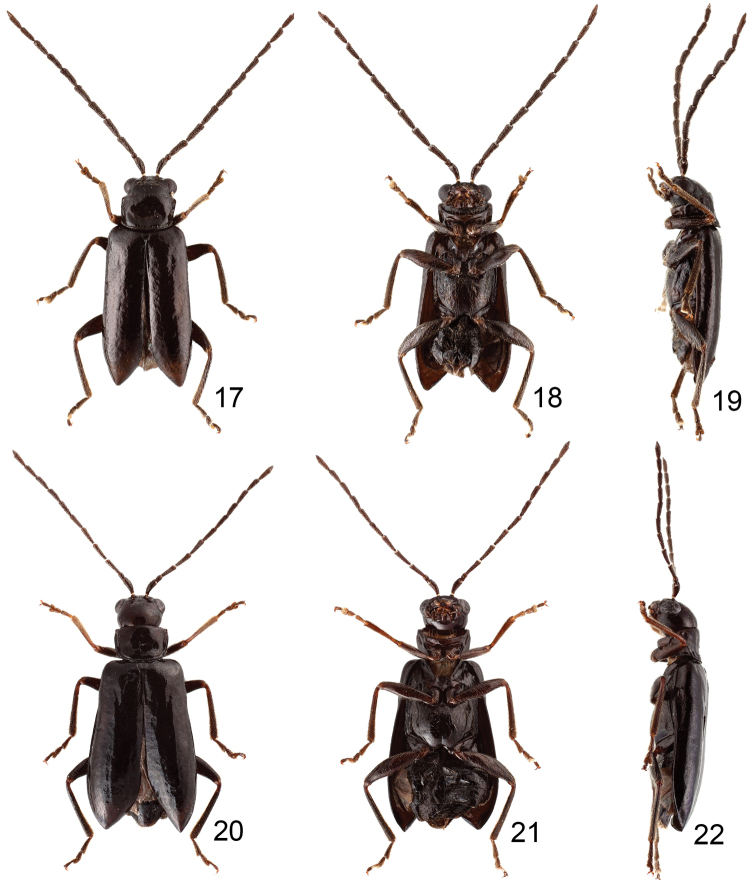
Habitus of *Jolibrotica chujoi*. **17** Male, dorsal view **18** ditto, ventral view **19** ditto, lateral view **20** Female, dorsal view **21** ditto, ventral view **22** ditto, lateral view.

**Figures 23–29.**
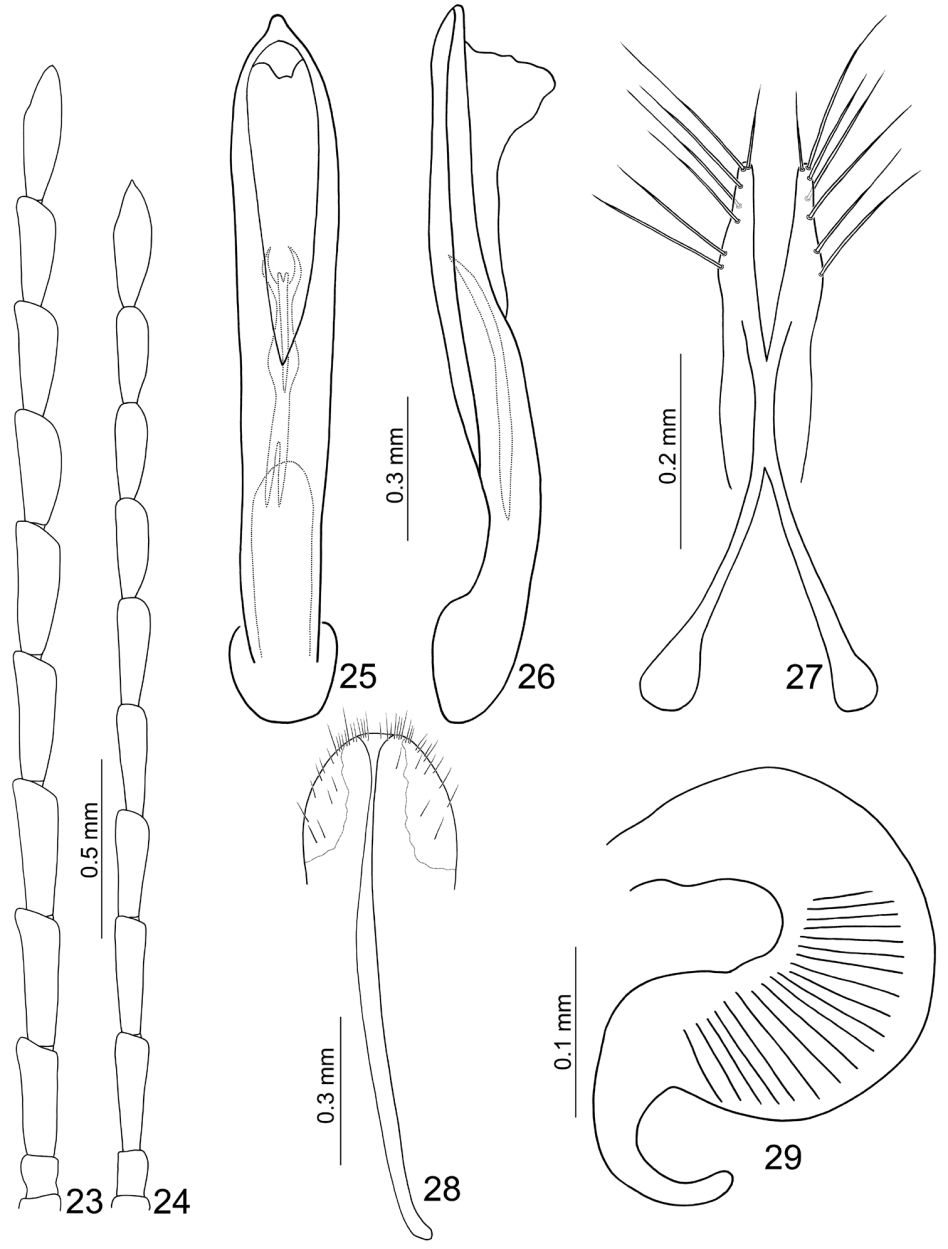
*Jolibrotica chujoi*. **23** Antenna male **24** Antenna, female **25** Penis, dorsal view **26** Penis, lateral view **27** Gonocoxae **28** Ventrite VIII **29** Spermatheca.

Length 3.2–3.8 mm, width 1.2 mm. Color blackish brown (Figs 17–19). Eyes small, distance between eyes 4.3 times wider than diameter of eye. Antenna (Fig. 23) filiform and long, as long as body size, ratio of length of antennomeres III to XI about 1.0 : 1.2 : 1.2 : 1.1 : 1.1 : 1.0 : 0.9 : 0.9 : 1.1; ratio of length to width from antennomere III to XI about 2.8 : 3.1 : 3.1 : 3.0 : 3.0 : 2.7 : 2.8 : 2.7 : 3.6. Pronotum quadrilateral; 1.47-1.54 times wider than long; widened anteriorly; disc moderately depressed behind middle. Elytra elongate, 1.66–1.69 times longer than wide; widest at apical 1/3. First tarsomeres normal. Abdominal ventrites without modification, ventrite V with apical margin truncate. Penis (Figs 25, 26) elongate, about 6.8 times longer than wide, apex rounded with small distinct tip, widest at apical 1/10, towards base gradually narrowed; tectum membranous; ventral surface with longitudinal median area membranous; extremely slender and slightly curved behind mddile at lateral view; internal sac with one longitudinal sclerite, apex forming inwards forked processes, base deeply bifurcate.

#### Females.

Length 4.1–4.3 mm, width 2.1–2.2 mm. Similar to male (Figs 20–22); antenna relatively slender than in male (Fig. 24), about 0.9 times as long as body, ratio of length of antennomeres III to XI about 1.0 : 1.0 : 0.9 : 0.9 : 0.9 : 0.8 : 0.8 : 0.8 : 1.1; ratio of length to width from antennomere III to XI about 3.6 : 3.6 : 3.2 : 3.2 : 3.2 : 3.0 : 3.0 : 2.9 : 3.3. Apical margin of ventrite V truncate. Gonocoxae (Fig. 27) slender, well separated from each other, combined together from apical 2/5 to 3/5; each gonocoxa with seven setae from apical 1/6 to apex. Ventrite VIII (Fig. 28) only laterally sclerotized; apical margin widely rounded, with several long setae near lateral margins, and several long and dense setae mixed along apical margin. Spermathecal receptaculum (Fig. 29) extremely swollen; pump extremely slender and curved; sclerotized spermathecal duct short and wide.

#### Distribution.

Endemic to southern Taiwan below 1500 m elevation (Fig. 15).

#### Host plant.

*Callicarpa kochiana* Makino (Verbenaceae).

### Haplosomoides shirozui

Taxon classificationAnimaliaColeopteraChrysomelidae

(Kimoto, 1969) comb. n.

Phyllobrotica shirozui Kimoto, 1969: 37; Wilcox 1973: 474; Kimoto 1989: 252; Kimoto 1991: 12; Takizawa et al. 1995: 12; Kimoto and Chu 1996: 72; Kimoto and Takizawa 1997: 303, 378; Beenen 2010: 487.

#### Type locality.

Taiwan: Chiayi county, Fenchihu [奮起湖], 23°30'22"N, 120°42'01"E, 1500 m.

#### Type material examined.

Holotype ♂ (KUEC), labeled: “(Taiwan) / Fenchihu, 1400m / Chiayi Hsien [p, w] // 12.IV.[h]1965[p] / T. Shirôzu [p, w] // Host: [p] / 食草標本 No. 6 [h, w] // Japan-U. S. / Co-op. Sci. / Programme [p, y] // *Phyllobrotica* / *shirozui* / Kimoto, n. sp, [h, w] // HOLOTYPE [p, r]”. Paratypes: 1♀ (KMNH): “(Taiwan) / Fenchihu, 1400m / Chiayi Hsien [p, w] // 12.IV[h]. 1965[p] / T. Shirôzu [p, w] // Host: [p] / 食草標本 No. 6 [h, w] // Japan-U. S. / Co-op. Sci. / Programme [p, y] // *Phyllobrotica* / *shirozui* / Kimoto, n. sp, [h, w] // PARATOPOTYPE [p, b]”; 1♂ (KMNH), same but without “Host: [p] / 食草標本 No. 6 [h, w]”; 1 ex., (KMNH): “(Taiwan) / Sungkang / Nantou Hsien [p, w] // 10.VI[h].1965[p] / T. Shirôzu [p, w] // *Phyllobrotica* / *shirozui* / Kimoto, n. sp, [h, w] // PARATOPOTYPE [p, b]”; 2♀♀, (KMNH): “(Taiwan) / Sungkang / Nantou Hsien [p, w] // 31. [h]V.1965[p] / T. Shirôzu [p, w] // *Phyllobrotica* / *shirozui* / Kimoto, n. sp, [h, w] // PARATYPE [p, b]”; 2♂♂ (KMNH): “(Taiwan) / Sungkang / Nantou Hsien [p, w] // 10. VI. [h]1965[p] / T. Shirôzu [p, w] // *Phyllobrotica* / *shirozui* / Kimoto, n. sp, [h, w] // PARATYPE [p, b]”; 1♂ (KMNH), same but with “PARATOPOTYPE [p, b]”; 1♀ (KMNH): “(Taiwan) / Sungkang / Nantou Hsien [p, w] // 29[h]. vi. 1965[p] / T. Yamasaki [p, w] // Japan-U. S. / Co-op. Sci. / Programme [p, y] // *Phyllobrotica* / *shirozui* / Kimoto, n. sp, [h, w] // PARATYPE [p, b]”; 1♀ (KMNH): “(Taiwan) / Sungkang, 2000m / --Tsifeng, 2300m / Nantou Hsien [p, w] // 29[h]. vi. 1965[p] / S. Kimoto [p, w] // Japan-U. S. / Co-op. Sci. / Programme [p, y] // *Phyllobrotica* / *shirozui* / Kimoto, n. sp, [h, w] // PARATYPE [p, b]”.

#### Additional specimens examined

(n = 247). **TAIWAN. Chiayi**: 1♂, Fenchihu, 25.V.2013, leg. W.-C. Liao (TARI); 2♂♂, Laichitashan, 19.III.2009, leg. H. Lee (TARI); **Hualien**: 1♂, 1♀, Tayuling, 9–16.VI.1980, leg. K. S. Lin & B. H. Chen (TARI); **Hsinchu**: 2♂♂, 3♀♀, Kuanwu, 1.V.2010, leg. M.-H. Tsou (TARI); **Kaoshiung**: 1♂, 1♀, Chungchihkuan, 17.IV.2012, leg. L.-P. Hsu (TARI); 2♂♂, 4♀♀, Erhchituan, 8.III.2013, leg. B.-X. Guo (TARI); 1♂, Shanping, 22.III.2014, leg. W.-C. Liao (TARI); 5♂♂, 4♀♀, Shihshan logging trail, 24.III.2009, leg. M.-H. Tsou (TARI); 3♂♂, 3♀♀, same data, S.-F. Yu leg. (JBCB); 13♂♂, 9♀♀, Tengchih, 2–5.VI.2008, leg. C.-F. Lee (TARI); 1♂, 2♀♀, Tona logging trail, 12.III.2013, leg. B.-X. Guo (TARI); **Nantou**: 1♀, Fenghuanshan, 9.III.2014, leg. J.-C. Chen (TARI); 1♂, 1♀, Meifeng, 10.V.1979, leg. K. C. Chou (TARI); 2♀♀, same locality, 17–22.VI.1979 (TARI); 1♂, 1♀, same locality, 20–22.VI.1979, leg. K. S. Lin & B. H. Chen (TARI); 1♀, same locality, 22–29.VI.1979 (TARI); 1♀, same locality, 27–29.VI.1979, leg. K. S. Lin & L. Y. Chou (TARI); 1♀, same locality, 2–4.VI.1980, leg. L. Y. Chou & C. C. Chen (TARI); 1♂, 2♀♀, same locality, 8.VI.1980, leg. K. S. Lin & B. H. Chen (TARI); 37♂♂, 43♀♀, same locality, 7–9.V.1981, leg. K. S. Lin & S. C. Lin (TARI); 6♂♂, 7♀♀, same locality, 24–26.VI.1981, leg. K. S. Lin & W. S. Tang (TARI); 4♂♂, 11♀♀, same locality, 22.V.1982, leg. L. Y. Chou (TARI); 4♀♀, same locality, 15.VII.1982, leg. S. C. Lin & C. N. Lin (TARI); 7♂♂, 6♀♀, same locality, 19–21.IV.1983, leg. K. C. Chou & S. P. Huang; 2♂♂, 1♀, same locality, 8–11.V.1984, leg. K. C. Chou & C. C. Pan (TARI); 2♀♀, Tatachia, 9.VI.2009, leg. C.-F. Lee (TARI); 3♂♂, 6♀♀, Tunyuan, 27.IV.2014, leg. M.-H. Tsou (TARI); **Pingtung**: 7♂♂, 4♀♀, Lilungshan, 8.III.2014, leg. J.-C. Chen (TARI); 1♂, Peitawushan, 17.II.2010, leg. S.-F. Yu (TARI); 2♂♂, same locality, 8.IV.2013, leg. Y.-T. Chung (TARI); 6♂♂, 1♀, same locality, 19.II.2014, leg. Y.-T. Chung (TARI); 3♂♂, 2♀♀, same locality, 22.IV.2014, leg. Y.-T. Chung (TARI); 1♂, 1♀, same locality, 8.V.2014, leg. Y.-T. Chung (TARI); 1♀, Tahanshan, 6.IV.2013, leg. W.-C. Liao (TARI); **Taichung**: 2♀♀, Anmashan, 7.VI.2010, leg. C.-F. Lee (TARI); 1♀, Wuling, 27–29.VI.1979, leg. K. S. Lin & L. Y. Chou (TARI); **Tainan**: 1♂, Meiling, 24.III.2011, leg. U. Ong (TARI); **Taitung**: 1♂, Liyuan, 29.III.2011, leg. C.-F. Lee (TARI).

#### Diagnosis.

Although *Haplosomoides shirozui* resembles *Haplosomoides changi* Lee, Bezděk & Staines, 2011 with the similarly peculiar shaped penis and absence of longitudinal ridge on the elytron, it can be easily recognized by its metallic blue elytra (pale in *Haplosomoides changi*, see Lee et al 2011).

#### Males.

**Figures 30–32.**
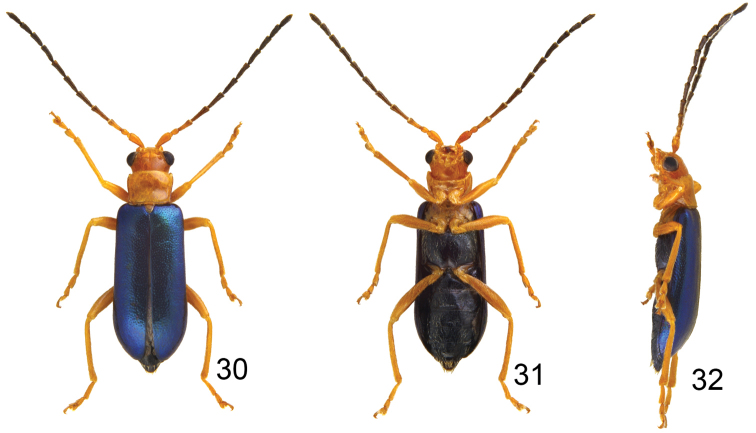
Habitus of *Haplosomoides shirozui*, male. **30** Dorsal view **31** Ventral view **32** Lateral view.

**Figures 33–39.**
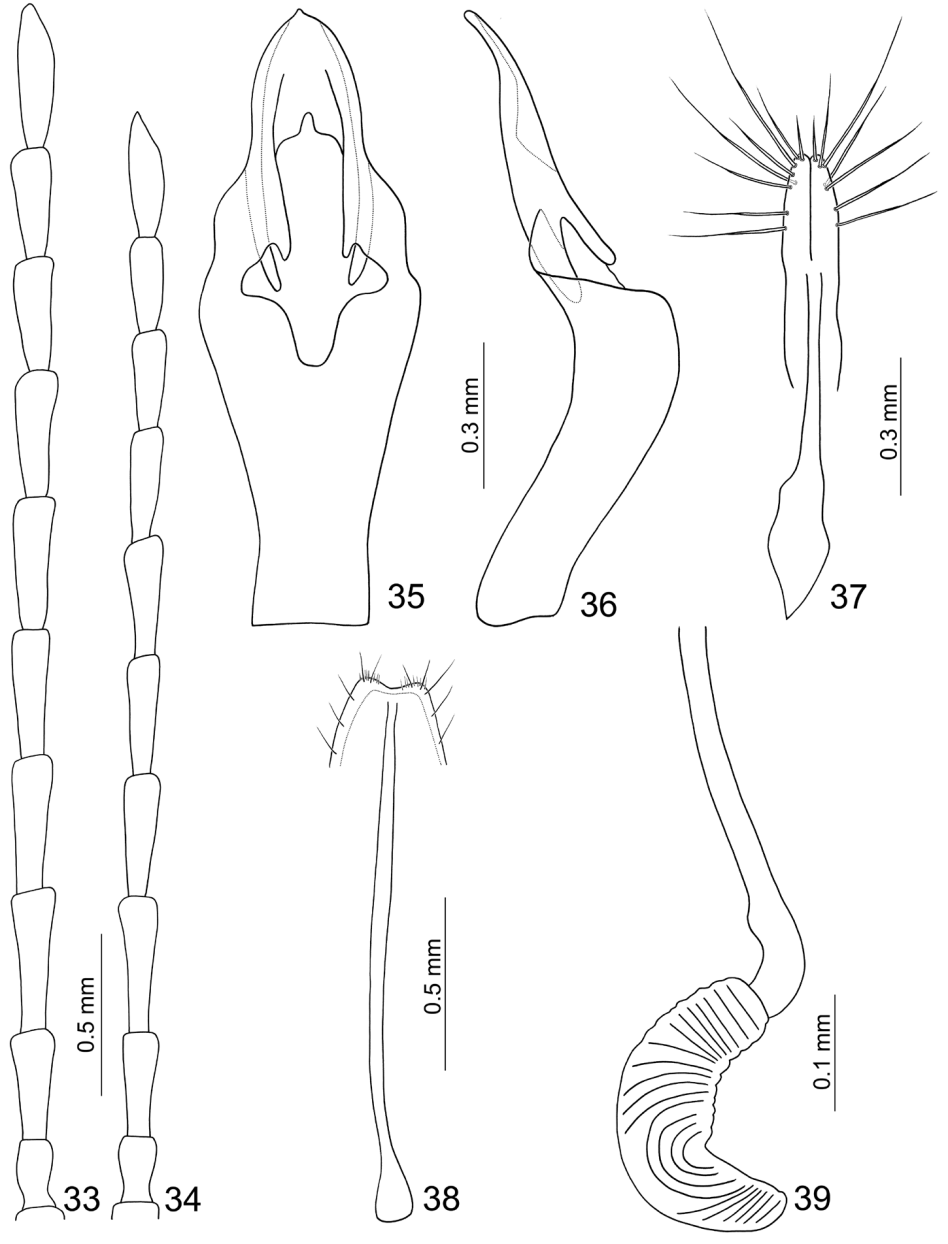
*Haplosomoides shirozui*. **33** Antenna male **34** Antenna, female **35** Penis, dorsal view **36** Penis, lateral view **37** Gonocoxae **38** Ventrite VIII **39** Spermatheca.

Length 4.1–5.1 mm, width 1.4–1.7 mm. Color yellowish-brown (Figs 30–32); antenna blackish-brown, three or four basal antennomeres paler; elytron metallically blue; metathoracic and abdominal ventrites black. Eyes extremely small, distance between eyes 3.7 times wider than diameter of eye. Antenna (Fig. 33) filiform and long, 0.9 times as long as body size, ratio of length of antennomeres III to XI about 1.0 : 1.3 : 1.3 : 1.2 : 1.2 : 1.2 : 1.0 : 1.0 : 1.3; ratio of length to width from antennomere III to XI about 2.6 : 3.3 : 3.4 : 3.2 : 3.3 : 3.1 : 2.8 : 2.7 : 3.9. Pronotum quadrilateral; 1.33–1.47 times wider than long; widened anteriorly; disc moderately depressed behind middle. Elytra elongate, 2.17–2.23 times longer than wide; parallel-sided. First tarsomeres normal. Abdominal ventrites without modification, ventrite V with apical margin truncate. Penis (Figs 35, 36) abruptly widened at middle, apex pointed, with median and wide groove from near apex to middle, central area membranous; ventral surface with longitudinal ridges close to lateral margin from apex to middle; moderately curved from lateral view.

#### Females.

Length 5.7–5.9 mm, width 2.0–2.2 mm. Similar to male; ratio of length of antennomeres III to XI about 1.0 : 1.3 : 1.2 : 1.1 : 1.1 : 1.1 : 0.9 : 0.9 : 1.2; ratio of length to width from antennomere III to XI about 2.9 : 3.8 : 3.4 : 3.3 : 3.3 : 3.2 : 2.7 : 2.8 : 3.9 (Fig. 34). Apical margin of ventrite V widely rounded. Gonocoxae (Fig. 37) slender, extremely close to each other from apex to apical 1/4, each gonocoxa with seven to eight setae from apical 1/6 to apex; basally combined from base to apical 1/4. Ventrite VIII (Fig. 38) with lateral and apical margin strongly sclerotized; apical margin emarginate, with several long setae along lateral margins, and one cluster of short setae at antero-lateral angles. Spermathecal receptaculum (Fig. 39) as wide as pump; pump hardly separated from rectptaculum, moderately curved; sclerotized spermathecal duct slender and extremely long.

#### Distribution.

Endemic to Taiwan. Although not as widespread as *Jolibrotica sauteri*, it is abundant locally in mountains at elevations between 1000 and 2500 m, extending north to Hsinchu County.

#### Host plant.

*Clerodendrum trichotomum* Thunb (Verbenaceae).

#### Discussion.

*Phyllobrotica shirozui* is transferred to *Haplosomoides* based on male abdomen simple (strongly modified in *Phyllobrotica*), pronotum with wide transverse depression in posterior half (pronotum regularly convex in *Phyllobrotica*) and elytral epipleura present (absent in *Phyllobrotica*). *Haplosomoides shirozui* belongs to *Haplosomoides annamita* species group as defined by Lee et al (2011) and in the structure of aedeagus it is very close to *Haplosomoides changi* Lee, Bezděk & Staines, 2011.

## Supplementary Material

XML Treatment for Jolibrotica

XML Treatment for Jolibrotica sauteri

XML Treatment for Jolibrotica chujoi

XML Treatment for Haplosomoides shirozui
